# CL22209, a standardized *Asparagus racemosus* root extract, demonstrates improved ovarian morphology, menstrual regularity, and metabolic parameters in women with polycystic ovary syndrome in a randomized, controlled trial

**DOI:** 10.29219/fnr.v69.13244

**Published:** 2025-12-23

**Authors:** Sridevi Kondamudi, Sravanthi Sadu, Sujatha Deva, Aishwarya Yalamanchi, Amulya Yalamanchi

**Affiliations:** 1 Department of Gynecology, Vijaya Sai Hospital, Vijayawada, India; 2 Department of Radiology, Vennela Scan Center and Diagnostics, Vijayawada, India; 3 Department of General Medicine, Orizin Endocrine Centre, Vijayawada, India

**Keywords:** Asparagus racemosus, hormone regulation, hypothalamic-pituitary-ovarian axis, menstrual regularity, polycystic ovary syndrome, broad-spectrum safety

## Abstract

**Background and objective:**

Polycystic ovary syndrome (PCOS) is a leading cause of infertility and metabolic dysfunction in women of reproductive age. Despite its high prevalence, current medical treatments are largely symptom-targeted and often prescribed off-label, highlighting the need for safer, integrative interventions. Botanical formulations with phytoestrogenic and insulin-sensitizing properties may represent a holistic therapeutic approach. The study aims to evaluate the clinical efficacy and safety of a standardized *Asparagus racemosus* root extract (CL22209) in women diagnosed with PCOS.

**Methods:**

A randomized, double-blind, placebo-controlled clinical trial (registration no: CTRI/2023/11/059457) was conducted in 60 women aged 20–35 years who were diagnosed with PCOS, as determined by the Rotterdam criteria. Participants received either CL22209 (100 mg daily) or a placebo for 84 consecutive days. The primary endpoint was the change in ovarian volume from the baseline. Secondary outcomes included ovarian cyst size and follicle number, menstrual cycle regularity, androgen-related manifestations, anthropometric indices, hormonal parameters, insulin sensitization, and safety.

**Results:**

After 84 days of supplementation, CL22209 significantly (*P* < 0.0001) reduced mean ovarian volume (20.98%), cyst size (40.97%), and follicle number (20.56%) as compared to placebo. The supplement showed exploratory indications of improved insulin sensitivity and hormonal profiles. Modest changes were also seen in menstrual patterns and anthropometric measures. CL22209 was well-tolerated over the study period.

**Conclusion:**

CL22209 was well-tolerated and demonstrated broad-spectrum efficacy in women diagnosed with PCOS, improving ovarian morphology, metabolic health, and androgen-mediated symptoms. Future studies with larger sample sizes and longer follow-up durations would help to further validate these findings and clarify CL22209’s role in the management of PCOS.

## Popular scientific summary

1. Polycystic ovary syndrome (PCOS) affects up to 15% of women of reproductive age, causing infertility, hormonal imbalance, and metabolic complications.
2. CL22209, a standardized extract of *Asparagus racemosus* (Shatavari), was tested in women diagnosed with PCOS in a randomized, placebo-controlled clinical trial.
3. Supplementation improved ovarian morphology, menstrual regularity, insulin sensitivity, and reduced hirsutism, acne, and central obesity.
4. CL22209 was well-tolerated and offers a promising plant-based therapy for holistic PCOS management.

Polycystic ovary syndrome (PCOS) is a prevalent and heterogeneous endocrine disorder that affects an estimated 5–15% of women of reproductive age (15–49 years) worldwide (1–3). It is one of the major causes of female infertility, implicated in approximately 70–80% of anovulatory infertility cases (4). The global burden of PCOS is steadily increasing, with higher prevalence reported among women in the Americas, South Asia, the Middle East, and Hispanic populations, potentially due to genetic susceptibility and environmental factors such as diet, sedentary lifestyle, and urbanization (5, 6). In addition to reproductive dysfunction, PCOS is associated with a broad spectrum of metabolic and psychological comorbidities, including insulin resistance (IR), type 2 diabetes, dyslipidaemia, cardiovascular risk, anxiety, and depression, making it a significant global public health concern in women.

The pathophysiology of PCOS is characterized by hyperandrogenism, IR, and ovulatory dysfunction (7–10). Hyperandrogenism triggers clinical manifestations such as menstrual irregularities, hirsutism, and acne (11). Moreover, IR contributes to hyperinsulinemia, dyslipidaemia, central obesity, and systemic low-grade inflammation, further aggravating hyperandrogenism (12, 13). Ovulatory dysfunction arises from disruptions in the hypothalamic–pituitary–ovarian (HPO) axis, characterized by increased luteinizing hormone (LH), reduced follicle-stimulating hormone (FSH), and elevated ovarian androgen production, leading to arrested follicular development (7, 10). Reactive oxygen species (ROS) and oxidative stress (OS) have also been implicated as drivers of PCOS pathology (14).

Clinically, women with PCOS most frequently report infertility, menstrual disturbances, excessive hair growth, and weight gain (15). Despite the high prevalence of PCOS, most available pharmacotherapies are not specifically used for its management. Instead, they are prescribed off-label and broadly address individual symptoms rather than targeting the syndrome comprehensively. Common approaches include oral contraceptives for menstrual regulation, insulin sensitizers (e.g. metformin, pioglitazone, glucagon-like peptide-1 agonists) for metabolic homeostasis, and antiandrogens (e.g. spironolactone, statins) for hirsutism and acne (16, 17). However, these interventions are often limited by adverse effects, including gastrointestinal discomfort, weight gain, mood disturbances, and cardiovascular risks (17, 18). Given these limitations, there is growing interest in integrative approaches combining traditional remedies with evidence-based evaluations.

Among Ayurvedic remedies, *Asparagus racemosus* (Shatavari) is well-recognized for its reproductive health benefits and is traditionally referred to as the ‘queen of herbs’ for women. Shatavari is rich in steroidal saponins (Shatavarins I–VI), flavonoids (rutin, hyperoside), mucilage, and alkaloids, which collectively support estrogenic modulation, reduce OS, and promote uterine–ovarian health (14, 19). Preclinical and limited clinical studies have demonstrated its potential in regulating hormonal imbalances, promoting folliculogenesis, and restoring ovulatory cycles (14, 19). However, the earlier investigations on Shatavari extracts for PCOS have limitations, including small sample sizes, lack of standardization, inconsistent methodologies, and inadequate mechanistic data.

CL22209 is a standardized root extract of *A. racemosus* containing not less than 15% total Shatavarins. Previous studies have shown its beneficial effects in improving menstrual health, reducing dysmenorrhea, and modulating gonadotropin levels in perimenopausal women by decreasing FSH and LH, while increasing 17β-estradiol (E2) and anti-Müllerian hormone (AMH) (19). We hypothesized that CL22209 supplementation would reduce ovarian volume and follicle count, decrease the LH:FSH ratio and total testosterone (TT) levels, increase sex hormone-binding globulin (SHBG), and enhance insulin sensitivity (Homeostatic Model Assessment for Insulin Resistance [HOMA-IR]) compared with placebo.

To evaluate this hypothesis, an 84-day randomized, double-blind, placebo-controlled clinical trial was conducted to explore the tolerability, efficacy, and mechanistic action of CL22209 in women diagnosed with PCOS. Key endpoints included changes in ovarian volume, cyst size and follicle number, menstrual regularity, hirsutism score, acne grade, anthropometric measures (body weight, waist and hip circumferences), IR, and serum endocrine factors. Additionally, safety evaluations were performed via blood biochemistry and urine parameter assessments. This integrative proof-of-concept clinical investigation demonstrates CL22209 supplementation as a plant-based, well-tolerated, and therapeutic strategy for PCOS management, addressing both endocrine and metabolic dysfunctions in women.

## Materials and methods

### Study material: standardized *Asparagus racemosus* root extract (CL22209)

CL22209 is a patent-pending botanical extract formulation derived from the tuberous roots of *A. racemosus* Willd. The raw plant material was purchased from the Morena district of Madhya Pradesh, India. Taxonomic authentication was conducted by a certified botanist, with identification confirmed against a validated herbarium reference. A voucher specimen has been archived (accession no. 6243) at the Chemiloids Life Sciences Research and Development Center, Aswaraopet, Telangana, India.

The botanical extract preparation process was described earlier (19). The final formulation consists of eight parts of botanical extract and two parts of pharmaceutical-grade excipients, ensuring homogeneity and a free-flowing powdered consistency. The formulation is standardized to contain not less than 15% total Shatavarins, as quantified using validated high-performance liquid chromatography (HPLC) methods (19). CL22209 was developed and manufactured under Current Good Manufacturing Practices (cGMP) at Laila Nutra Private Limited, Vijayawada, India. The present study utilized a batch of CL22209 (No. LPP23100195), produced in October 2023, with a 2-year shelf life.

### Protocol registration and ethics approval

The study protocol was reviewed and approved (ECR/564/Inst/AP/2014/RR-20) by the Institutional Ethics Committee of Yalamanchi Hospital, Vijayawada, Andhra Pradesh, India. This trial was registered (CTRI/2023/11/059457) with the Clinical Trials Registry – India (CTRI), New Delhi, India. The study was conducted during the period spanning 21 November 2023 to 11 June 2024, following the ethical principles outlined in the International Council for Harmonization – Good Clinical Practice (ICH-GCP) guidelines, as well as the Indian Council of Medical Research (ICMR) and the Ministry of Ayurveda, Yoga & Naturopathy, Unani, Siddha and Homoeopathy (AYUSH) guidelines. The risks and benefits of the study were explained, and written informed consent was obtained from all subjects before initiating any study-related procedure.

### Enrolment, sample size, and power calculation

A total of 60 sexually active female subjects (age: 20–35 years; body mass index (BMI): 22–29 kg/m²), were enrolled in this study. All participants were clinically diagnosed with PCOS based on the Rotterdam criteria (6) and recruited following inclusion and exclusion criteria (Supplementary Table S1).

The sample size calculation was based on a two-sided *t*-test with a significance level of α = 0.05 (5%). The sample size for this randomized, placebo-controlled clinical trial was determined based on the primary endpoint of mean change in ovarian volume from baseline. Parameters for estimation were derived from an earlier investigation (20), which reported a mean difference of 2.2 cm³ with a common standard deviation (SD) of 2.5 cm³ between intervention and control groups. This trial was statistically powered for the primary endpoint (ovarian volume). All secondary endpoints, including hormonal, metabolic, and anthropometric measures, were considered exploratory. A two-sided *t*-test at a 5% significance level (α = 0.05) with 90% statistical power indicated that 30 participants per group were required to detect a statistically relevant difference.

### Randomization and blinding

Participants were randomly assigned in a 1:1 ratio to receive either CL22209 (*n* = 30) or a placebo (*n* = 30). Allocation concealment was maintained using a centralized randomization system. The randomization sequence was generated by an independent statistician using the SAS PROC PLAN procedure with block randomization to ensure balanced allocation between treatment arms (21). Each participant was assigned a unique identification number linked to a pre-coded bottle of either CL22209 or placebo (a 1:1 blend of brown dextrin and maltodextrin). The capsules were visually and taste-wise indistinguishable. The allocation codes were securely maintained by the independent statistician and were not accessible to the investigators, study staff, or participants until the study database was locked.

### Intervention and follow-up

**Fig. 1:**
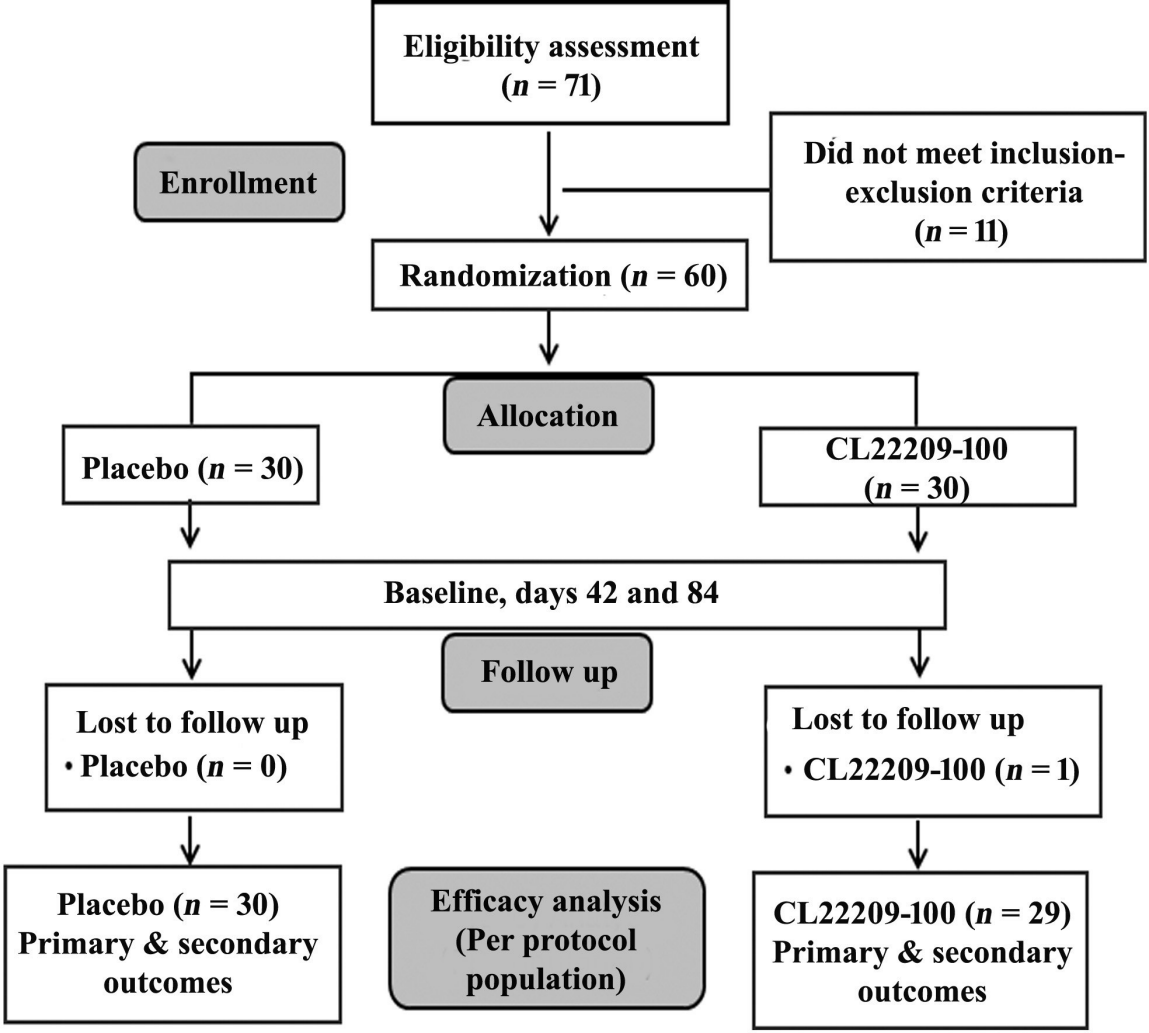
Consolidated Standards of Reporting Trials (CONSORT)-compliant flow diagram of participant enrolment, allocation, follow-up, and analysis.

Participants received one capsule daily, containing either CL22209 (100 mg) or a matching placebo, administered orally in the morning after breakfast over a period of 84 consecutive days. The investigational product (IP) was encapsulated in size 1 hard gelatine capsules and dispensed in coded high-density polyethylene (HDPE) bottles. The study was structured into four scheduled visits: (1) visit 1: Screening, (2) visit 2 (day 1): Randomization, (3) visit 3 (day 42): Midpoint follow-up, and (4) visit 4 (day 84): Completion of study assessment (Fig. 1).

Participants were instructed to maintain their usual diet and lifestyle and avoid any medications or supplements that could interfere with study outcomes. However, their diet and daily physical activities were not regulated. Compliance was monitored through daily subject diaries, in which participants recorded capsule intake, any concurrent medication, and any adverse events (AE). The principal investigator (PI) reviewed and endorsed these logs at regular intervals.

At each follow-up visit, all participants returned unused capsules, which were reconciled with the IP accountability log to confirm adherence. Attendance and compliance were carefully recorded to ensure accurate accountability. The PI and study coordinators reinforced adherence protocols during all visits. Additionally, physical health assessments and safety evaluations (including vital signs and general physical examinations) were conducted at screening and during each follow-up visit to ensure participant well-being and monitor for AE.

### Efficacy assessments

#### Ovarian volume, ovarian cyst size, and number of follicles

Ovarian volume, cyst size, and follicle count were assessed using a transvaginal ultrasound system (Model: Voluson E8, GE Healthcare, Bengaluru, India, and Philips Epic 7G, USO1480190, Bothell, WA), equipped with an 8 MHz transvaginal probe. All ultrasound examinations were conducted in a private clinical setting, following informed consent from each participant. To ensure anatomical accuracy, alignment with the utero-ovarian ligament was confirmed for optimal visualization of each ovary. Both transverse and sagittal planes were utilized to scan the ovaries comprehensively from the inner to outer edges. The assessments included: (1) Ovarian volume in cm^3^ (calculated using the prolate ellipsoid formula: length × width × height × 0.523), (2) Total follicle count, and (3) Maximum cyst diameter (mm) (22). Licensed sonographers performed all imaging following standardized protocols for the evaluation of polycystic ovarian morphology.

#### Assessment of menstrual cycle regularity

Menstrual cycle regularity is defined as the duration between two consecutive menstrual bleeding episodes (intermenstrual interval). Participants documented the onset of menstrual bleeding in their study diaries and compliance cards during the intervention period. These data were reviewed by the investigators at each follow-up visit to evaluate improvements in cycle regularity.

#### Assessment of modified Ferriman–Gallwey and Global Acne Grading System scores

Hirsutism was evaluated using the modified Ferriman–Gallwey (mFG) scoring system**,** which assesses terminal hair growth across nine body areas: upper lip, chin, chest, upper abdomen, lower abdomen, upper back, lower back, upper arms, and thighs (23). Each area was scored from 0 (no terminal hair**)** to 4 (extensive terminal hair), and the scores were summed to yield a total mFG score. Based on ethnic-specific evaluations, a cut-off mFG score of ≥ 5 was considered indicative of hirsutism in the South Asian population (24). The participants’ hair removal practices were not recorded during the intervention.

Acne severity was assessed using the Global Acne Grading System (GAGS), which evaluates six anatomical locations – forehead, right and left cheek, nose, chin, and chest and upper back, each assigned a factor based on surface area and sebaceous gland activity (25). Lesions were graded by type (comedones, papules, pustules, nodules), and the scores were multiplied by the area factor to obtain a total GAGS score. Acne severity was categorized as mild (1–18)**,** moderate (19–30)**,** severe (31–38)**,** and very severe (**>**38**)**.

#### Measurement of body weight, waist, and hip circumferences

Body weight was measured using a digital weighing machine (Phoenix, Bengaluru, India), with participants wearing light clothing and no shoes. Waist circumference was measured at the midpoint between the lower margin of the last palpable rib and the upper edge of the iliac crest, using a non-elastic measuring tape with an insertion buckle to maintain consistent tension. Hip circumference was measured at the level of the maximum protrusion of the buttocks, just below the iliac crest.

#### Serum endocrine factors and insulin resistance measurements

Fasting venous blood samples were collected during days 3–5 of the menstrual cycle at both baseline and post-intervention to assess circulatory hormone levels. Blood samples were drawn by trained phlebotomists, following standard procedure. The clear serum samples (3,000 rpm for 15 min, 4°C) were stored at –80°C in aliquots. FSH, LH, TT, and SHBG were measured using a biochemistry analyser, Cobas 6000 (Roche Healthcare, Basel, Switzerland).

LH (Elecsys Ref# 11732234122), FSH (Elecsys FSH Ref#11775863122, TT (Elecsys Testosterone II Ref#05200067190), and SHBG (Elecsys SHBG Ref# 03052001190) were measured using electrochemiluminescence immunoassay kits following the assay procedures provided by the vendor (Roche Diagnostics GmbH, Baden‑Württemberg, Germany). The lower limits of detection for LH, FSH, TT, and SHBG were 0.100 mIU/mL, 0.100 mIU/mL, 0.025 ng/mL, and 0.350 nmol/L, respectively. All assays were performed in a National Accreditation Board for Testing and Calibration Laboratories (NABL) certified facility, Tenet Diagnostics, Banjara Hills, Hyderabad, India (certificate no. MC-2977, valid 10 October 2023 to 09 October 2025).

IR was estimated using the HOMA-IR, calculated from fasting glucose and insulin concentrations according to the following formula (26).

HOMA-IR = [glucose (mg/dL) × serum insulin (mUI/L)/405]

#### Safety measurements

Comprehensive safety evaluations were performed at screening and at the end of the intervention period (day 84) to ensure participant well-being and identify any treatment-emergent adverse effects. Vital signs, haematology, fasting blood biochemistry, urinalysis, and systemic AE were assessed in accordance with the GCP guidelines. Haematological analysis included red blood cell (RBC) count, haemoglobin (Hb), platelet count, total and differential leukocyte counts, and erythrocyte sedimentation rate (ESR).

A detailed serum biochemistry panel was conducted to assess metabolic, hepatic, renal, electrolyte, and cardiovascular–muscular function, measuring: fasting blood glucose (FBG), total cholesterol, high-density lipoprotein (HDL), low-density lipoprotein (LDL), very low-density lipoprotein (VLDL) triglycerides; alanine transaminase (ALT), aspartate aminotransferase (AST), alkaline phosphatase (ALP), total bilirubin, albumin; creatinine, blood urea nitrogen (BUN), uric acid; sodium, potassium, and creatine kinase (CK). Urinalysis parameters included pH, colour, specific gravity, and the presence of protein, glucose, and RBCs.

#### Statistical analysis

A total of 30 participants were enrolled per treatment arm, accommodating an anticipated 5% attrition rate (~1 participant per group). The sample size determination was based on a two-sided *t*-test with a 5% significance level (α = 0.05) and 90% statistical power, assuming a mean difference of 2.2 and a typical SD of 2.5 in the change from baseline ovarian volume, as reported earlier (20).

All efficacy analyses were conducted within the per-protocol (PP) population, and results are expressed as mean ± SD. One subject in the CL22209 group was lost to follow-up, the missing vales were handled using baseline observation carried forward (BOCF) method. Descriptive statistics summarized demographic and baseline variables, clinical laboratory results, and vital signs, with paired *t*-tests applied for within-group assessments. In PP population, efficacy outcomes were evaluated using analysis of covariance (ANCOVA) for both intragroup and intergroup comparisons. In the intention-to-treat (ITT) population, the comparison analyses were performed using ANCOVA followed by Bonferroni-Holm correction (data presented in Supplementary Tables S2–S7). All statistical analyses were performed using SAS^®^ software, version 9.4 (SAS Institute Inc., Cary, NC, USA).

## Results

### Baseline demographics and participant compliance

In this trial, 60 women (age: 20–35 years; BMI: 22–29 kg/m²) were enrolled based on clinical symptoms consistent with PCOS. The participants were classified as having PCOS phenotype D, characterized by oligo- and/or anovulation in combination with polycystic ovarian morphology. Participants were randomly assigned to receive either 100 mg of standardized *A. racemosus* root extract (CL22209) or matching placebo capsules once daily over a period of 84 consecutive days. Baseline demographic characteristics were comparable between groups (Table 1). At baseline, serum levels of LH, FSH, SHBG, TT, and HOMA-IR in the placebo group were 12.13 ± 5.19 mIU/mL, 6.43 ± 1.83 mIU/mL, 35.52 ± 13.12 nmol/L, 0.64 ± 0.46 ng/mL, and 3.19 ± 0.41, respectively. The corresponding values in the CL22209 group were 10.66 ± 3.55 mIU/mL, 6.17 ± 1.96 mIU/mL, 35.58 ± 15.14 nmol/L, 0.65 ± 0.39 ng/mL, and 3.29 ± 0.41, respectively. Between-group comparisons indicated no statistically significant differences in these hormonal or metabolic parameters at baseline (Table 7).

**Table 1:** . Demographic characteristics

Parameters	Mean ± SD	*P*-value (vs. placebo)	95% CI vs. placebo
**Age (years)**			
Placebo (*n* = 30)	27.2 ± 4.3	-	-
CL22209 (*n* = 30)	28.3 ± 4.3	0.3560	–1.19, 3.27
**Body mass index (BMI) (kg/m^2^)**			
Placebo (*n* = 30)	26.9 ± 1.5	-	-
CL22209 (*n* = 30)	26.8 ± 1.6	0.7396	–0.69, 0.95
**Height (** ***m*** **)**			
Placebo (*n* = 30)	1.5 ± 0.0	-	-
CL22209 (*n* = 30)	1.5 ± 0.0	0.6513	–0.00, 0.02
**Body weight (kg)**			
Placebo (*n* = 30)	63.5 ± 4.7	-	-
CL22209 (*n* = 30)	62.9 ± 4.7	0.6224	–1.83, 3.03

Between the groups comparison was analysed using ANCOVA.

A PP analysis was performed on 59 participants, as one subject in the CL22209 group was lost to follow-up. Adherence to the IP was high in both groups, with mean compliance rates of 98.6% for placebo and 98.4% for CL22209.

### CL22209 supplementation reduced ovarian volume

At the end of the intervention period, right ovarian volume was significantly lower in the CL22209 group as compared to the placebo group (*P* < 0.0001), while left ovarian volume was significantly reduced at both 42 and 84 days of the intervention as compared to placebo (*P* < 0.0001) (Table 2). Mean ovarian volume, calculated as the average of the right and left ovarian volumes, was also significantly reduced in the CL22209 group at both 42 and 84 days of treatment, as compared with baseline and placebo (*P* < 0.0001) (Table 2).

**Table 2:** . Assessment of ovarian volume

Group	Time of Evaluation	Mean ± SD (cm^3^)	Change from baseline Mean ± SD	*P*-value (vs. baseline)	*P*-value (vs. placebo)	95% CI vs. placebo	Cohen’s d vs. placebo
**Right ovarian volume**							
Placebo (*n* = 30)	Baseline	11.83 ± 1.66	-	-	-	**-**	**-**
	Day 42	11.19 ± 1.88	–0.64 ± 2.19	0.0183	-	**-**	**-**
	Day 84	11.43 ± 1.61	–0.40 ± 1.16	0.0422	-	**-**	**-**
CL22209 100 mg (*n* = 29)	Baseline	12.00 ± 1.70	-	-	0.7084	–0.71, 1.05	0.10
	Day 42	10.67 ± 2.14	–1.33 ± 1.30	<0.0001	0.1267	–0.53, 1.57	0.26
	Day 84	9.06 ± 1.56	–2.94 ± 1.51	<0.0001	<0.0001	1.54, 3.20	1.50
**Left ovarian volume**							
Placebo (*n* = 30)	Baseline	11.01 ± 1.84	-	-	-	**-**	**-**
	Day 42	11.37 ± 1.76	0.36 ± 0.84	0.1538	-	**-**	**-**
	Day 84	11.07 ± 1.79	0.06 ± 1.63	0.9564	-	**-**	**-**
CL22209 100 mg (*n* = 29)	Baseline	11.06 ± 2.25	-	-	0.9269	–1.02, 1.12	0.02
	Day 42	9.15 ± 2.02	–1.91 ± 1.63	<0.0001	<0.0001	1.23, 3.21	1.17
	Day 84	8.73 ± 2.10	–2.33 ± 1.72	<0.0001	<0.0001	1.32, 3.36	1.20
**Mean ovarian volume (left and right ovaries)**							
Placebo (*n* = 30)	Baseline	11.42 ± 1.38	-	-	-	-	**-**
	Day 42	11.28 ± 1.78	–0.14 ± 1.17	0.4389	-	-	**-**
	Day 84	11.25 ± 1.38	–0.17 ± 1.11	0.2860	-	-	**-**
CL22209 100 mg (*n* = 29)	Baseline	11.53 ± 1.54	-	-	0.7696	–0.65, 0.87	0.08
	Day 42	9.91 ± 1.66	–1.62 ± 1.20	<0.0001	<0.0001	0.47, 2.27	0.80
	Day 84	8.89 ± 1.50	–2.64 ± 1.22	<0.0001	<0.0001	1.61, 3.11	1.64

A *P*-value < 0.05 indicates significance in intragroup (vs. baseline) or intergroup comparison (CL22209 vs. placebo) analysed using ANCOVA followed by Bonferroni–Holm correction.

In the placebo group, the changes in the right ovarian volumes on days 42 (*P* = 0.0183) and 84 (*P* = 0.0422) of the study were significant versus baseline; however, the changes in the left ovarian volume and mean ovarian volumes were not significant at the end of the study (Table 2). Overall. at the end of the trial, 18 participants (62.07%) in the CL22209 group showed ovarian volume of ≤ 10 cm³, compared with only two participants (6.67%) in the placebo group.

### CL22209 supplementation reduced ovarian cyst size and follicle count

Post-trial, the CL22209-supplemented group exhibited significant reductions in mean ovarian cyst size as well as for both the right and left ovaries as compared to baseline and the placebo group (*P* < 0.0001). In the placebo group, these changes were not significant (vs. baseline) (Table 3).

**Table 3:** . Assessment of mean ovarian cyst size and number of ovarian follicles

Group	Time of evaluation	Mean ± SD	Change from baseline Mean ± SD	*P*-value (vs. baseline)	*P*-value (vs. placebo)	95% CI (vs. placebo)	Cohen’s d vs. placebo
**Cyst size (Right ovary, mm)**							
Placebo (*n* = 30)	Base line	6.17 ± 1.28	-	-	-	-	-
	Day 42	6.07 ± 1.20	–0.10 ± 0.82	0.8023	-	-	-
	Day 84	6.01 ± 1.40	–0.16 ± 1.02	0.6367	-	-	-
CL22209 100 mg (*n* = 29)	Base line	5.92 ± 1.19	-	-	0.4553	–0.39, 0.89	0.20
	Day 42	4.14 ± 1.57	–1.78 ± 1.71	<0.0001	<0.0001	1.20, 2.66	1.38
	Day 84	3.38 ± 1.32	–2.54 ± 1.52	<0.0001	<0.0001	1.92, 3.34	1.93
**Cyst size (Left ovary, mm)**							
Placebo (*n* = 30)	Baseline	7.08 ± 1.13	-	-	-	-	-
	Day 42	6.72 ± 1.31	–0.36 ± 1.11	0.1362	-	-	-
	Day 84	6.83 ± 1.29	–0.25 ± 1.15	0.4819	-	-	-
CL22209 100 mg (*n* = 29)	Baseline	6.99 ± 1.02	-	-	0.7487	–0.47, 0.65	0.08
	Day 42	5.66 ± 1.32	–1.33 ± 1.56	<0.0001	0.0009	0.37, 1.75	0.81
	Day 84	4.21 ± 1.62	–2.78 ± 1.76	<0.0001	<0.0001	1.86, 3.38	1.79
**Mean cyst size (left and right ovaries, mm)**							
Placebo (*n* = 30)	Baseline	6.62 ± 0.99	-	-	-	-	-
	Day 42	6.40 ± 1.08	–0.23 ± 0.78	0.1702	-	-	-
	Day 84	6.42 ± 1.08	–0.21 ± 0.81	0.3823	-	-	-
CL22209 100 mg (*n* = 29)	Baseline	6.45 ± 0.83	-	-	0.4821	–0.31, 0.65	0.19
	Day 42	4.90 ± 1.16	–1.56 ± 1.06	<0.0001	<0.0001	0.92, 2.08	1.34
	Day 84	3.79 ± 1.26	–2.66 ± 1.18	<0.0001	<0.0001	2.02, 3.24	2.24
**Number of follicles (Right ovary)**							
Placebo (*n* = 30)	Baseline	19.37 ± 3.10	-	-	-	-	-
	Day 42	18.10 ± 3.25	–1.27 ± 2.15	0.0032	-	-	-
	Day 84	18.50 ± 4.27	–0.87 ± 3.18	0.1297	-	-	-
CL22209 100 mg (*n* = 29)	Baseline	19.28 ± 3.62	-	-	0.9117	–1.67, 1.85	0.03
	Day 42	17.38 ± 3.11	–1.90 ± 2.26	<0.0001	0.2561	–0.94, 2.38	0.23
	Day 84	13.86 ± 2.50	–5.41 ± 3.61	<0.0001	<0.0001	2.81, 6.47	1.33
**Number of follicles (Left ovary)**							
Placebo (*n* = 30)	Base line	18.67 ± 3.36	-	-	-	-	-
	Day 42	18.93 ± 3.27	0.27 ± 1.41	0.6884	-	-	-
	Day 84	17.30 ± 3.52	–1.37 ± 3.20	0.0068	-	-	-
CL22209 100 mg (*n* = 29)	Base line	18.38 ± 3.36	-	-	0.7142	–1.46, 2.04	0.09
	Day 42	16.28 ± 3.18	–2.10 ± 3.31	<0.0001	0.0002	0.97, 4.33	0.82
	Day 84	14.59 ± 2.68	–3.79 ± 4.30	<0.0001	0.0012	1.07, 4.35	0.87
**Average number of follicles (left and right ovaries)**							
Placebo (*n* = 30)	Baseline	19.02 ± 2.96	-	-	-	-	-
	Day 42	18.52 ± 2.84	–0.50 ± 1.50	0.1385	-	-	-
	Day 84	17.90 ± 3.03	–1.12 ± 2.10	0.0039	-	-	-
CL22209 100 mg (*n* = 29)	Baseline	18.83 ± 2.87	-	-	0.7841	–1.33, 1.71	0.07
	Day 42	16.83 ± 2.50	–2.00 ± 2.20	<0.0001	0.0020	0.29, 3.09	0.63
	Day 84	14.22 ± 1.94	–4.60 ± 2.90	<0.0001	<0.0001	2.35, 5.01	1.45

A *P*-value < 0.05 indicates significance in intragroup (vs. baseline) or intergroup comparison (CL22209 vs. placebo) analysed using ANCOVA followed by the Bonferroni–Holm correction.

CL22209 supplementation significantly reduced (*P* < 0.0001 vs. baseline) the number of follicles in both ovaries. Compared with placebo, the CL22209 group showed fewer follicles in the right ovary on day 84 (*P* < 0.0001) and in the left ovary on both days 42 (*P* = 0.0002) and 84 (*P* = 0.0012) of supplementation. Mean follicle counts (combined from both ovaries) also declined significantly on days 42 and 84 in the CL22209 group (*P* < 0.0001 vs. baseline). These counts were considerably lower than those in the placebo group at both days 42 (*P* = 0.0020) and 84 (*P* < 0.0001) of treatment (Table 3). At baseline, 16 participants in the placebo group and 13 participants in the CL22209 group had ≥ 20 follicles per ovary. By the end of the study, this number had decreased to 8 in the placebo group, while none of the participants in the CL22209 group exhibited ≥ 20 follicles per ovary.

In the placebo group, post-trial, the number of follicles in the left ovary (*P* = 0.0068), and the average number of follicles (*P* = 0.0039) were reduced as compared to baseline (Table 3).

### CL22209 supplementation improved menstrual cycle regularity

Following 84 days of supplementation, participants in the CL22209 group exhibited a significant improvement in menstrual cycle regularity, as evidenced by a reduction in the interval between consecutive menstrual bleeding episodes as compared to baseline. Post-trial, the mean intermenstrual interval had decreased by 12.11% (*P* < 0.0001 vs. baseline) in the CL22209 group, as compared with a 6.05% reduction (*P* =0.0031) in the placebo group (Table 4). In the between-groups comparison, these changes were not significant; however, the magnitude of change was greater in the CL22209 group, indicating a possible improvement in menstrual cycle regulation in the study volunteers.

**Table 4:** . Assessment of regularity in menstrual cycles

Group	Time of evaluation	Duration between two consecutive bleedings (days) (mean ± SD)	Change from baseline Mean ± SD	*P*-value (vs. baseline)	*P*-value (vs. placebo)	95% CI vs. placebo	Cohen’s d vs. placebo
Placebo (*n* = 30)	Baseline	45.73 ± 8.41	-	-	-	-	-
	Day 42	48.60 ± 5.16	2.87 ± 11.71	0.5845	-	-	-
	Day 84	42.97 ± 8.56	–2.77 ± 3.65	0.0031	-	-	-
CL22209 100 mg (*n* = 29)	Baseline	48.69 ± 6.26	-	-	0.0943	–0.92, 6.84	0.40
	Day 42	48.21 ± 4.09	–0.48 ± 7.87	0.4328	0.8657	–2.04, 2.82	0.08
	Day 84	42.79 ± 6.62	–5.90 ± 7.17	<0.0001	0.1122	–3.82, 4.18	0.02

A *P*-value < 0.05 indicates significance in intragroup (vs. baseline) or intergroup comparison (CL22209 vs. placebo) analysed using ANCOVA followed by the Bonferroni–Holm correction.

### Effects of CL22209 supplementation on hirsutism and acne severity

In the CL22209 group, mFG scores were significantly reduced (*P* < 0.0001) on days 42 and 84 of supplementation as compared to baseline. In comparison with the placebo group, the mFG scores in the CL22209 group were also significant (day 42: *P* = 0.0001 and day 84: *P* < 0.0001) (Table 5).

**Table 5:** . Modified Ferriman–Gallwey Questionnaire for Hirsutism and Global Acne Grading System scores

Group	Time of Evaluation	Mean ± SD	Change vs. baseline Mean ± SD	*P*-value (vs. baseline)	*P*-value (vs. placebo)	95% CI vs. placebo	Cohen’s d vs. placebo
**Modified Ferriman–Gallwey Questionnaire for Hirsutism (mFG) scores**							
Placebo (*n* = 30)	Baseline	6.97 ± 1.35	-	-	-	-	-
	Day 42	6.47 ± 1.41	–0.50 ± 1.80	0.0427	-	-	-
	Day 84	6.60 ± 1.38	–0.37 ± 1.16	0.1106	-	-	-
CL22209 100 mg (*n* = 29)	Baseline	7.00 ± 1.58	-	-	0.9318	–0.74, 0.79	0.02
	Day 42	5.10 ± 1.18	–1.90 ± 1.54	<0.0001	0.0001	0.69, 2.05	1.05
	Day 84	3.79 ± 0.82	–3.21 ± 1.84	<0.0001	<0.0001	2.22, 3.40	2.48
**Global Acne Grading System (GAGS) scores**							
Placebo (*n* = 30)	Baseline	8.93 ± 2.97	-	-	-	-	-
	Day 42	8.43 ± 3.28	–0.50 ± 1.50	0.0584	-	-	-
	Day 84	8.27 ± 3.60	–0.67 ± 2.23	0.0218	-	-	-
CL22209 100 mg (*n* = 29)	Baseline	9.10 ± 2.51	-	-	0.7904	–1.27, 1.61	0.06
	Day 42	6.90 ± 1.72	–2.21 ± 1.26	<0.0001	<0.0001	0.16, 2.90	0.58
	Day 84	4.66 ± 1.32	–4.45 ± 1.68	<0.0001	<0.0001	2.19, 5.03	1.33

A *P*-value < 0.05 indicates significance in intragroup (vs. baseline) or intergroup comparison (CL22209 vs. placebo) analysed using ANCOVA followed by the Bonferroni–Holm correction.

Similarly, GAGS scores decreased significantly (*P* < 0.0001) from baseline in the CL22209 group at both days 42 and 84 of intervention. Between-group analysis also showed significantly lower GAGS scores (*P* < 0.0001 vs. placebo) in the CL22209 group on days 42 and 84 of supplementation (Table 5).

### Effect of CL22209 supplementation on anthropometric measures

In the CL22209 group, body weight significantly decreased (*P* < 0.0001) from baseline at both days 42 (1.86%) and 84 (2.89%) of the study; these reductions were significantly greater (*P* < 0.0001 vs. placebo) at both time points (Table 6).

**Table 6:** . Anthropometric measurements

Groups	Time of evaluation	Mean ± SD	Change from baseline Mean ± SD	*P*-value (vs. baseline)	*P*-value (vs. placebo)	95% CI vs. placebo	Cohen’s d vs. placebo
**Body weight (kg)**							
Placebo (*n* = 30)	Baseline	63.53 ± 4.72	-	-	-	-	-
	Day 42	63.32 ± 4.54	–0.21 ± 0.71	0.1875	-	-	-
	Day 84	63.29 ± 4.56	–0.24 ± 0.67	0.1554	-	-	-
CL22209 100 mg (*n* = 29)	Baseline	62.90 ± 4.75	-	-	0.6046	–1.84, 3.10	0.13
	Day 42	61.73 ± 4.91	–1.17 ± 0.98	<0.0001	<0.0001	–0.87, 4.05	0.34
	Day 84	61.08 ± 5.05	–1.82 ± 1.30	<0.0001	<0.0001	–0.29, 4.72	0.46
**Waist circumference (cm)**							
Placebo (*n* = 30)	Baseline	78.61 ± 5.64	-	-	-	-	-
	Day 42	78.43 ± 5.67	–0.18 ± 0.75	0.2507	-	-	-
	Day 84	78.38 ± 5.68	–0.23 ± 0.63	0.0733	-	-	-
CL22209 100 mg (*n* = 29)	Baseline	79.84 ± 4.53	-	-	0.3486	–1.44, 3.90	0.24
	Day 42	79.07 ± 4.58	–0.78 ± 0.94	<0.0001	0.0115	–2.05, 3.33	0.12
	Day 84	78.12 ± 4.47	–1.72 ± 0.92	<0.0001	<0.0001	–2.41, 2.93	0.05
**Hip circumference (cm)**							
Placebo (*n* = 30)	Baseline	97.03 ± 5.67	-	-	-	-	-
	Day 42	96.92 ± 5.72	–0.11 ± 1.07	0.5537	-	-	-
	Day 84	96.88 ± 5.54	–0.15 ± 1.45	0.4909	-	-	-
CL22209 100 mg (*n* = 29)	Baseline	97.00 ± 5.59	-	-	0.9801	–2.91, 2.97	0.01
	Day 42	96.19 ± 5.57	–0.81 ± 0.95	<0.0001	0.0123	–2.21, 3.67	0.13
	Day 84	95.36 ± 5.50	–1.64 ± 0.86	<0.0001	<0.0001	–1.36, 4.39	0.28

A *P*-value < 0.05 indicates significance in intragroup (vs. baseline) or intergroup comparison (CL22209 vs. placebo) analysed using ANCOVA followed by Bonferroni-Holm correction.

Waist circumference was significantly reduced (*P* < 0.0001 vs. baseline) in the CL22209 group on days 42 (0.98%) and 84 (2.15%) of treatment. Compared to the placebo group, the reductions were significantly greater in the CL22209 group on days 42 (*P* = 0.0115) and 84 (*P* < 0.0001) (Table 6).

Similarly, in the CL22209 group, hip circumference decreased significantly (*P* < 0.0001 vs. baseline) by 0.84 and 1.69% at 42 and 84 days of supplementation, respectively. Between-groups comparisons revealed substantially greater reductions in the CL22209 group (vs. placebo) on day 42 (*P* = 0.0123) and day 84 (*P* < 0.0001) (Table 6).

In the placebo group, body weight, waist, and hip circumferences showed minimal changes from baseline, with reductions of 0.33 and 0.38%, 0.23 and 0.29%, and 0.11 and 0.15% on days 42 and 84, respectively. These changes were not statistically significant vs. baseline (Table 6).

### Effect of CL22209 supplementation on serum endocrine factors and insulin sensitivity

Post-trial, the CL22209 group exhibited a significant increase in serum FSH levels compared to baseline (*P* = 0.0041). No significant within-group changes in LH were observed in either group. However, at the end of the study, the LH-to-FSH ratio in the CL22209 group was significantly decreased (*P* = 0.0002 vs. baseline; *P* = 0.0034 vs. placebo) (Table 7).

**Table 7:** . Serum endocrine factors and HOMA-IR

Groups	Time of evaluation	Mean ± SD	Change from baseline Mean ± SD	*P*-value (vs. baseline)	*P*-value (vs. placebo)	95% CI (vs. placebo)	Cohen’s d vs. placebo
**Luteinizing hormone (LH) (mIU/mL)**							
Placebo (*n* = 30)	Baseline	12.13 ± 5.19	-	-	-	-	-
	Day 84	12.53 ± 5.95	0.40 ± 5.29	0.2898	-	-	-
CL22209 100 mg (*n* = 29)	Baseline	10.66 ± 3.55	-	-	0.1737	–0.86, 3.79	0.33
	Day 84	11.41 ± 5.30	0.75 ± 5.73	0.4839	0.8018	–1.82, 4.06	0.20
**Follicle stimulating hormone (FSH) (mIU/mL)**							
Placebo (*n* = 30)	Baseline	6.43 ± 1.83	-	-	-	-	-
	Day 84	6.82 ± 2.87	0.38 ± 2.45	0.5136	-	-	-
CL22209 100 mg (*n* = 29)	Baseline	6.17 ± 1.96	-	-	0.5857	–0.73, 1.25	0.14
	Day 84	8.04 ± 4.92	1.87 ± 4.63	0.0041	0.1050	–0.87, 3.31	0.30
**LH to FSH ratio**							
Placebo (*n* = 30)	Baseline	1.85 ± 0.42	-	-	-	-	-
	Day 84	1.83 ± 0.45	–0.02 ± 0.54	0.7531	-	-	-
CL22209 100 mg (*n* = 29)	Baseline	1.77 ± 0.41	-	-	0.4568	–0.86, 3.79	0.19
	Day 84	1.49 ± 0.44	–0.28 ± 0.52	0.0002	0.0034	0.11, 0.57	0.76
**Sex Hormone Binding Globulin (SHBG) (nmol/L)**							
Placebo (*n* = 30)	Baseline	35.52 ± 13.12	-	-	-	-	-
	Day 84	31.69 ± 11.32	–3.83 ± 14.68	0.0958	-	-	-
CL22209 100 mg (*n* = 29)	Baseline	35.58 ± 15.14	-	-	0.9881	–0.14, 0.29	0.00
	Day 84	41.75 ± 13.74	6.17 ± 15.20	0.0011	0.0006	3.51, 16.61	0.80
**Total testosterone (ng/mL)**							
Placebo (*n* = 30)	Baseline	0.64 ± 0.46	-	-	-	-	-
	Day 84	0.65 ± 0.43	0.01 ± 0.22	0.9280	-	-	-
CL22209 100 mg (*n* = 29)	Baseline	0.65 ± 0.39	-	-	0.963	–0.21, 0.23	0.02
	Day 84	0.43 ± 0.28	–0.21 ± 0.36	<0.0001	0.0006	0.03, 0.41	0.61
**Homeostatic Model Assessment for Insulin Resistance (HOMA-IR)**							
Placebo (*n* = 30)	Baseline	3.19 ± 0.41	-	-	-	-	-
	Day 42	3.33 ± 0.52	0.15 ± 0.63	0.3683	-	-	-
	Day 84	3.28 ± 0.54	0.09 ± 0.65	0.8140	-	-	-
CL22209 100 mg (*n* = 29)	Baseline	3.29 ± 0.41	-	-	0.3720	–0.11, 0.31	0.24
	Day 42	3.02 ± 0.48	–0.27 ± 0.57	0.0038	0.0073	0.05, 0.57	0.62
	Day 84	2.82 ± 0.42	–0.47 ± 0.57	<0.0001	<0.0001	0.21, 0.71	0.95

A *P*-value < 0.05 indicates significance in intragroup (vs. baseline) or intergroup comparison (CL22209 vs. placebo) analysed using ANCOVA followed by Bonferroni–Holm correction.

Serum SHBG levels increased significantly in the CL22209 group (*P* = 0.0011 vs. baseline) and were also significantly higher than placebo after 84 days of supplementation (*P* = 0.0006) (Table 7).

At the completion of the study, the CL22209 supplementation significantly reduced serum TT levels (*P* < 0.0001 vs. baseline; *P* = 0.0006 vs. placebo) (Table 7).

As an exploratory observation, insulin sensitivity, assessed using HOMA-IR, showed significant improvement (vs. baseline) in the CL22209 group on days 42 (*P* = 0.0038) and 84 (*P* < 0.0001) of supplementation. Between-group comparisons revealed significantly greater improvements in the CL22209 group on days 42 (*P* = 0.0073) and 84 (*P* < 0.0001) compared to the placebo group (Table 7).

### Safety assessments and AEs

CL22209 was well-tolerated with no clinically significant changes in haematological or biochemical parameters, including liver enzymes, markers of renal function and cardiovascular function, lipid profile, and complete blood counts (Supplementary Table S8). The laboratory values remained within reference ranges throughout the study. During the intervention, in the placebo group, three participants reported experiencing either headaches, nasal congestion, or a sore throat. However in the CL22209 group, one participant reported a cough, and another reported a runny nose (allergic rhinitis). All five AE were mild and transient; the participants recovered without requiring any pharmacological interventions.

## Discussion

PCOS is a highly prevalent endocrine–metabolic disorder, affecting an estimated 5–15% of women of reproductive age and contributing to nearly 70% of anovulatory infertility cases globally (4). Its pathophysiology is complex, encompassing hyperandrogenism, ovulatory dysfunction, and polycystic ovarian morphology, frequently accompanied by metabolic dysfunctions including IR, dyslipidaemia, and central adiposity (13, 16, 27). Morphological features such as ovarian enlargement, excessive follicle number, and stromal hypertrophy are directly linked to anovulation (28), and menstrual irregularity remains a core diagnostic and quality-of-life concern.

This study evaluated the efficacy of CL22209, a standardized *A. racemosus* root extract, in women diagnosed with PCOS. Supplementation over a consecutive 84-day period resulted in significant improvements in ovarian morphology, including reductions in ovarian volume, cyst size, and follicle count, as well as improved menstrual cycle regularity. These findings align with an earlier study supporting the role of *A. racemosus* in menstrual regulation, enhancement of endometrial thickness, and reduction of hirsutism in PCOS (29), as well as observed benefits in menstrual regularities in perimenopausal women (19). However, a small but statistically significant reduction in right ovarian volume was also observed in the placebo group, likely reflecting natural variability in ovarian morphology or regression to the mean. Given the lack of consistent changes in other ovarian or hormonal parameters, this finding is not considered clinically meaningful.

Furthermore, the observed decrease in follicle number suggests that CL22209 may modulate folliculogenesis by rebalancing HPO axis signalling. Dysregulated GnRH pulsatility in PCOS triggers LH hypersecretion over FSH, promoting theca cell androgen excess and impairing granulosa cell aromatase activity (28, 30). By lowering the LH to FSH ratio, CL22209 could facilitate granulosa cell aromatization of androgens into estradiol, supporting dominant follicle selection and ovulatory competence (31). The observed clinical improvements with CL22209 may be partially explained by its proposed phytoestrogenic activity (32), and modulation of the HPO axis (33). However, these mechanisms were not directly investigated in the present study and remain theoretical, warrant confirmation through dedicated mechanistic studies.

In the present study, although the participants in the CL22209 group demonstrated a greater reduction in the intermenstrual interval compared to the placebo, the between-group difference was not statistically significant. This finding may reflect natural cycle variability or a placebo effect, as participation in a clinical trial itself can positively influence health behaviours, stress levels, and self-monitoring, which are known to impact menstrual regularity. Moreover, some women with PCOS experience intermittent spontaneous ovulation, which could partially explain the improvements observed in the placebo group.

The phytoestrogenic steroidal saponins (Shatavarins) present in CL22209 may further contribute by interacting with estrogen receptors (ERα, ERβ), exerting estrogenic or selective estrogen receptor modulator (SERM)-like effects (14). These actions enhance central feedback regulation of gonadotropin secretion, attenuate ovarian stromal hypertrophy, and limit excessive recruitment of small follicles, a hallmark of PCOS morphology. Furthermore, the improvement in cycle regularity observed in the CL22209-supplemented subjects, although not significantly different from the placebo, may indicate improved ovulatory function. This improvement is critical for enhancing fertility and metabolic balance. Specifically, during the 84-day intervention, no major AE were reported, and unaltered haematology and total blood biochemistry parameters, including safety markers for liver, muscle, heart, kidney, and lung, collectively suggest that CL22209 supplementation was well-tolerated. However, a long-term safety and sustained efficacy of CL22209 remain to be further evaluated.

CL22209 supplementation significantly reduced HOMA-IR values**,** indicating enhanced insulin sensitivity in the enrolled volunteers. This finding is noteworthy given that IR is central to the pathophysiology of PCOS, where hyperinsulinemia augments ovarian and adrenal androgen production and suppresses hepatic SHBG synthesis (34, 35). By improving insulin sensitivity, CL22209 may indirectly normalize androgen levels and restore ovarian function. Hyperinsulinemia is known to synergize with LH in ovarian theca cells, amplifying androgen biosynthesis via cytochrome P450c17 upregulation, which contributes to a hyperandrogenic intraovarian environment that impairs follicular maturation (36). Attenuation of IR following CL22209 supplementation could therefore lower intraovarian androgen concentrations, improving follicular microenvironment and supporting ovulation.

Consistent with this mechanistic framework, the participants receiving CL22209 exhibited increased serum SHBG levels and reduced TT concentrations, two critical indicators of hormonal normalization in PCOS. Elevated SHBG reduced the pool of bioavailable androgens, thereby mitigating the hyperandrogenic drive, while reductions in circulating testosterone directly alleviated clinical manifestations such as hirsutism and acne in the CL22209-supplemented subjects. These endocrine and metabolic improvements support the prior evidence from *Trigonella foenum*-graecum extract-based interventions, which demonstrated enhanced SHBG levels, reduced androgen burden, and improved clinical outcomes in women with PCOS (37, 38).

Anthropometric outcomes further supported the overall findings, with statistically significant reductions observed in body weight, waist circumference, and hip circumference following CL22209 supplementation. However, the magnitude of these changes (~2–3%) was modest and falls below the thresholds typically considered clinically meaningful for weight loss (39). As dietary intake and physical activity were not objectively monitored in the present study, these reductions may not conclusively be attributed to the intervention. Accordingly, the observed anthropometric improvements should be interpreted as preliminary. However, these outcomes are relevant given the established associations of central adiposity with IR, cardiovascular disease, and dyslipidaemia. Abdominal obesity, measured by waist circumference, is a stronger predictor of IR, metabolic syndrome, and cardiovascular dysfunction than BMI alone (40, 41). Mechanistically, reductions in body weight and waist-hip circumference may be mediated by the effects on energy metabolism and fat oxidation, as hypothesized for other phytoestrogens and polyphenol-rich botanicals (42–44).

Taken together, CL22209 demonstrated a broad-spectrum efficacy by improving ovarian morphology, restoring menstrual regularity, alleviating androgen-mediated symptoms, enhancing metabolic competence, and supporting weight management, without any major AE. These observations highlight the therapeutic potential of standardized *A. racemosus* extract as a plant-based, integrative approach for managing PCOS. Future studies with larger cohorts and longer durations, incorporating dietary and activity monitoring as well as mechanistic endpoints, are warranted to validate these findings and strengthen the evidence for the sustained benefits of CL22209 in women with PCOS.

## Conclusion

In summary, this randomized, double-blind, placebo-controlled study provides preliminary evidence that CL22209 supplementation may offer beneficial effects on ovarian morphology, hormonal balance, and metabolic parameters in women with PCOS. Although the outcomes are encouraging, larger, multi-centre, longer-duration trials incorporating dietary and activity monitoring, mechanistic assessments, and long-term safety evaluations are warranted to confirm these preliminary results and establish the efficacy and durability of CL22209 in PCOS management.

## Supplementary Material



## Data Availability

The data supporting the published observations are available on request.
