# Supplementary material for: CL22209, a standardized Asparagus racemosus root extract, demonstrates improved ovarian morphology, menstrual regularity, and metabolic parameters in women with polycystic ovary syndrome in a randomized, controlled trial
Source: Food Nutr Res. 2025 Dec 23;69:10.29219/fnr.v69.13244. doi: 10.29219/fnr.v69.13244 (PMC12767677; doi:10.29219/fnr.v69.13244)
Supplement: Supplementary file 1 [file FNR-69-13244-s1.docx]

Supplementary Table S1: Inclusion-exclusion criteria

| Inclusion Criteria | Exclusion Criteria |
| --- | --- |
| - Women aged 20–35 years with BMI 22–29 kg/m². | - Medical history of cardiovascular, renal or liver disorders, endocrine-related diseases; active gallbladder disease; gynaecologic or breast surgery within 6 months. |
| - Diagnosed with PCOS per Rotterdam criteria. | - Use of hormonal therapy, metformin, or herbal supplements within 3 months. |
| - Oligo/anovulation in past year with FSH 1–10 U/L and normal estradiol (E2). | - Uncontrolled hypertension (SBP ≥160 mmHg or DBP ≥100 mmHg), Fasting blood glucose >125 mg/dL |
| - Polycystic ovarian morphology: ovarian volume ≥10 cm³ and/or ≥20 follicles per ovary. | - History of breast, endometrial, or gynaecologic cancer, or other malignancy within 5 years. |
| - Able to provide written informed consent and comply with study procedures. | - Pregnancy, lactation, or hypersensitivity to investigational product. |
| - Sexually active with non-hormonal contraception. | - Positive screening for hepatitis B/C, HIV, or syphilis. |
| - Normal vital signs, ECG, and laboratory tests. | - High alcohol intake (>2 drinks/day), recreational drug use, or psychiatric drug dependence. |
|  | - Participation in another clinical trial within 30 days. |

Supplementary Table S2. Assessment of ovarian volume (intention-to-treat analysis)

| Group | Time of Evaluation | Mean ± SD  (cm^3^) | Change from baseline Mean ± SD | *p*-value  (vs. baseline) | *p*-value  (vs. placebo) | 95% CI vs. placebo |
| --- | --- | --- | --- | --- | --- | --- |
| Right ovarian volume | | | | | |  |
| Placebo  (n=30) | Baseline | 11.83 ± 1.66 | - | - | - | **-** |
|  | Day 42 | 11.19 ± 1.88 | -0.64 ± 2.19 | 0.0221 | - | **-** |
|  | Day 84 | 11.43 ± 1.61 | -0.40 ± 1.16 | 0.0572 | - | **-** |
| CL22209  100 mg (n=30) | Baseline | 11.80 ± 2.01 | - | - | 0.9396 | -0.92, 0.98 |
|  | Day 42 | 10.51 ± 2.28 | -1.29 ± 1.30 | <0.0001 | 0.1029 | -0.40, 1.76 |
|  | Day 84 | 8.95 ± 1.64 | -2.84 ± 1.57 | <0.0001 | <0.0001 | 1.64, 3.32 |
| Left ovarian volume | | | | | |  |
| Placebo  (n=30) | Baseline | 11.01 ± 1.84 | - | - | - | **-** |
|  | Day 42 | 11.37 ± 1.76 | 0.36 ± 0.84 | 0.1391 | - | **-** |
|  | Day 84 | 11.07 ± 1.79 | 0.06 ± 1.63 | 0.8859 | - | **-** |
| CL22209  100 mg (n=30) | Baseline | 10.86 ± 2.47 | - | - | 0.7620 | -0.98, 1.28 |
|  | Day 42 | 9.01 ± 2.12 | -1.85 ±1 .64 | <0.0001 | <0.0001 | 1.35, 3.37 |
|  | Day 84 | 8.60 ± 2.17 | -2.25 ± 1.74 | <0.0001 | <0.0001 | 1.44, 3.50 |
| Mean ovarian volume (left and right ovaries) | | | | | |  |
| Placebo  (n=30) | Baseline | 11.42 ± 1.38 | - | - | - | - |
|  | Day 42 | 11.28 ± 1.78 | -0.14 ± 1.17 | 0.4627 | - | - |
|  | Day 84 | 11.25 ± 1.38 | -0.17 ± 1.11 | 0.3475 | - | - |
| CL22209  100 mg (n=30) | Baseline | 11.33 ± 1.88 | - | - | 0.8085 | -0.76, 0.94 |
|  | Day 42 | 9.76 ± 1.82 | -1. 57 ± 1.22 | <0.0001 | <0.0001 | 0.59, 2.45 |
|  | Day 84 | 8.78 ± 1.60 | -2.55 ± 1.29 | <0.0001 | <0.0001 | 1.70, 3.24 |

A *p*-value < 0.05 indicates significance in intragroup (vs. baseline) or intergroup comparison (CL22209 vs. placebo) analyzed using ANCOVA followed by Bonferroni-Holm correction.

Supplementary Table S3. Assessment of mean ovarian cyst size and number of ovarian follicles (intention-to-treat analysis)

| Group | Time of evaluation | Mean ± SD | Change from baseline Mean ± SD | *p*-value  (vs. baseline) | *p*-value  (vs. placebo) | 95% CI  (vs. placebo) |
| --- | --- | --- | --- | --- | --- | --- |
| Cyst size (Right ovary, mm) | | | | | | |
| Placebo  (n=30) | Base line | 6.17 ± 1.28 | - | - | - | - |
|  | Day 42 | 6.07 ± 1.20 | -0.10 ± 0.82 | 0.8129 | - | - |
|  | Day 84 | 6.01 ± 1.40 | -0.16 ± 1.02 | 0.6600 | - | - |
| CL22209  100 mg (n=30) | Base line | 5.95 ± 1.18 | - | - | 0.5210 | -0.42, 0.86 |
|  | Day 42 | 4.23 ± 1.63 | -1.72 ± 1.71 | <0.0001 | <0.0001 | 1.10, 2.58 |
|  | Day 84 | 3.50 ± 1.46 | -2.45 ± 1.57 | <0.0001 | <0.0001 | 1.77, 3.25 |
| Cyst size (Left ovary, mm) | | | | | | |
| Placebo  (n=30) | Baseline | 7.08 ± 1.13 | - | - | - | - |
|  | Day 42 | 6.72 ± 1.31 | -0.36 ± 1.11 | 0.1468 | - | - |
|  | Day 84 | 6.83 ± 1.29 | -0.25 ± 1.15 | 0.5150 | - | - |
| CL22209  100 mg (n=30) | Baseline | 7.02 ± 1.02 | - | - | 0.8467 | -0.50, 0.62 |
|  | Day 42 | 5.73 ± 1. 36 | -1.29 ± 1.55 | <0.0001 | 0.0017 | 0.30, 1.68 |
|  | Day 84 | 4.33 ±1.74 | -2.69 ± 1.80 | <0.0001 | <0.0001 | 1.71, 3.29 |
| Mean Cyst size (left and right ovaries, mm) | | | | | | |
| Placebo  (n=30) | Baseline | 6.62 ± 0.99 | - | - | - | - |
|  | Day 42 | 6.40 ± 1.08 | -0.23 ± 0.78 | 0.1808 | - | - |
|  | Day 84 | 6.42 ± 1.08 | -0.21 ± 0.81 | 0.4253 | - | - |
| CL22209  100 mg (n=30) | Baseline | 6.49 ± 0.84 | - | - | 0.5850 | -0.34, 0.60 |
|  | Day 42 | 4.98 ± 1.24 | -1.50 ± 1.08 | <0.0001 | <0.0001 | 0.82, 2.02 |
|  | Day 84 | 3.92 ± 1.41 | -2.57 ± 1.25 | <0.0001 | <0.0001 | 1.85, 3.15 |
| Number of follicles (Right ovary) | | | | | |  |
| Placebo  (n=30) | Baseline | 19.37 ± 3.10 | - | - | - | - |
|  | Day 42 | 18.10 ± 3.25 | -1.27 ± 2.15 | 0.0032 | - | - |
|  | Day 84 | 18.50 ± 4.27 | -0.87 ± 3.18 | 0.1383 | - | - |
| CL22209  100 mg (n=30) | Baseline | 19.33 ± 3.58 | - | - | 0.9670 | -1.69, 1.77 |
|  | Day 42 | 17.50 ± 3.13 | -1.83 ± 2.25 | <0.0001 | 0.3146 | -1.05, 2.25 |
|  | Day 84 | 14.10 ± 2.78 | -5.23 ± 3.68 | <0.0001 | <0.0001 | 2.54, 6.26 |
| Number of follicles (Left ovary) | | | | | |  |
| Placebo  (n=30) | Base line | 18.67 ± 3.36 | - | - | - | - |
|  | Day 42 | 18.93 ± 3.27 | 0.27 ± 1.41 | 0.7149 | - | - |
|  | Day 84 | 17.30 ± 3.52 | -1.37 ± 3.20 | 0.0071 | - | - |
| CL22209  100 mg(n=30) | Base line | 18.43 ± 3.32 | - | - | 0.7627 | -1.49, 1.97 |
|  | Day 42 | 16.40 ± 3.20 | -2.03 ± 3.27 | <0.0001 | 0.0003 | 0.86, 4.20 |
|  | Day 84 | 14.77 ± 2.81 | -3.67 ± 4.28 | <0.0001 | 0.0027 | 0.88, 4.18 |
| Average number of follicles (left and right ovaries) | | | | | |  |
| Placebo  (n=30) | Baseline | 19.02 ± 2.96 | - | - | - | - |
|  | Day 42 | 18.52 ± 2.84 | -0.50 ± 1.50 | 0.1379 | - | - |
|  | Day 84 | 17.90 ± 3.03 | -1.12 ± 2.10 | 0.0053 | - | - |
| CL22209  100 mg (n=30) | Baseline | 18.88 ± 2.83 | - | - | 0.8440 | -1.37, 1.64 |
|  | Day 42 | 16.95 ± 2.55 | -1.93 ± 2.19 | <0.0001 | 0.0035 | 0.18, 2.96 |
|  | Day 84 | 14.43 ± 2.22 | -4.45 ± 2.98 | <0.0001 | <0.0001 | 2.10, 4.84 |

A *p*-value < 0.05 indicates significance in intragroup (vs. baseline) or intergroup comparison (CL22209 vs. placebo) analyzed using ANCOVA followed by Bonferroni-Holm correction.

Supplementary Table S4. Assessment of regularity in menstrual cycles (intention-to-treat analysis)

| Group | Time of Evaluation | Duration between two consecutive bleedings  (days)  (mean ± SD) | Change from baseline  Mean ± SD | *p*-value  (vs. baseline) | *p*-value  (vs. placebo) | 95% CI vs. placebo |
| --- | --- | --- | --- | --- | --- | --- |
| Placebo  (n=30) | Baseline | 45.73 ± 8.41 | - | - | - | - |
|  | Day 42 | 48.60 ± 5.16 | 2.87 ± 11.71 | 0.4196 | - | - |
|  | Day 84 | 42.97 ± 8.56 | -2.77 ± 3.65 | 0.0029 | - | - |
| CL22209  100 mg (n=30) | Baseline | 48.57 ± 6.19 | - | - | 0.1065 | -0.98, 6.66 |
|  | Day 42 | 48.10 ± 4.06 | -0.47 ± 7.74 | 0.4445 | 0.9713 | -1.90, 2.90 |
|  | Day 84 | 42.87 ± 6.52 | -5.70 ± 7.13 | <0.0001 | 0.1381 | -3.83, 4.03 |

A *p*-value < 0.05 indicates significance in intragroup (vs. baseline) or intergroup comparison (CL22209 vs. placebo) analyzed using ANCOVA followed by Bonferroni-Holm correction.

Supplementary Table S5. Modified Ferriman-Gallwey Questionnaire for Hirsutism and Global Acne Grading System scores (intention-to-treat analysis)

| Group | Time of Evaluation | Mean ± SD | Change vs. baseline  Mean ± SD | *p*-value  (vs. baseline) | *p*-value  (vs. placebo) | 95% CI vs. placebo |
| --- | --- | --- | --- | --- | --- | --- |
| Modified Ferriman-Gallwey Questionnaire for Hirsutism (mFG) scores | | | | | | |
| Placebo  (n=30) | Baseline | 6.97 ± 1.35 | - | - | - | - |
|  | Day 42 | 6.47 ± 1.41 | -0.50 ± 1.80 | 0.0455 | - | - |
|  | Day 84 | 6.60 ± 1.38 | -0.37 ± 1.16 | 0.1331 | - | - |
| CL22209  100 mg (n=30) | Baseline | 7.00 ± 1.55 | - | - | 0.9305 | -0.72, 0.78 |
|  | Day 42 | 5.17 ± 1.21 | -1.83 ± 1.56 | <0.0001 | 0.0003 | 0.62, 1.98 |
|  | Day 84 | 3.90 ± 0.99 | -3.10 ± 1.90 | <0.0001 | <0.0001 | 2.08, 3.32 |
| Global Acne Grading System (GAGS) scores | | | | | | |
| Placebo  (n=30) | Baseline | 8.93 ± 2.97 | - | - | - | - |
|  | Day 42 | 8.43 ± 3.28 | -0.50 ± 1.50 | 0.0606 | - | - |
|  | Day 84 | 8.27 ± 3.60 | -0.67 ± 2.23 | 0.0306 | - | - |
| CL22209  100 mg (n=30) | Base line | 9.20 ± 2.52 | - | - | 0.6736 | -1.15, 1.69 |
|  | Day 42 | 7.07 ± 1.93 | -2.13 ± 1.31 | <0.0001 | 0.0001 | -0.03, 2.75 |
|  | Day 84 | 4.90 ± 1.86 | -4.30 ± 1.84 | <0.0001 | <0.0001 | 1.89, 4.85 |

A *p*-value < 0.05 indicates significance in intragroup (vs. baseline) or intergroup comparison (CL22209 vs. placebo) analyzed using ANCOVA followed by Bonferroni-Holm correction.

Supplementary Table S6: Anthropometric measurements (intention-to-treat analysis)

| Groups | Time of Evaluation | Mean ± SD | Change from baseline  Mean ± SD | *p*‐value (vs. baseline) | *p*‐value (vs. placebo) | 95% CI vs. placebo |
| --- | --- | --- | --- | --- | --- | --- |
| **Body weight (kg)** | | | | | | |
| Placebo  (n= 30) | Baseline | 63.53 ± 4.72 | - | - | - | - |
|  | Day 42 | 63.32 ± 4.54 | -0.21 ± 0.71 | 0.1887 | - | - |
|  | Day 84 | 63.29 ± 4.56 | -0.24 ± 0.67 | 0.1705 | - | - |
| CL22209  100mg (n=30) | Baseline | 62.93 ± 4.67 | - | - | 0.6202 | -1.83, 3.03 |
|  | Day 42 | 61.81 ± 4.84 | -1.13 ± 0.99 | <0.0001 | 0.0001 | -0.92, 3.94 |
|  | Day 84 | 61.17 ± 4.99 | -1.76 ± 1.32 | <0.0001 | <0.0001 | -0.35, 4.59 |
| **Waist Circumference (cm)** | | | | | | |
| Placebo  (n= 30) | Baseline | 78.61 ± 5.64 | - | - | - | - |
|  | Day 42 | 78.43 ± 5.67 | -0.18 ± 0.75 | 0.2596 | - | - |
|  | Day 84 | 78.38 ± 5.68 | -0.23 ± 0.63 | 0.1019 | - | - |
| CL22209  100mg (n=30) | Baseline | 80.45 ± 5.55 | - | - | 0.1846 | -1.05, 4.73 |
|  | Day 42 | 79.70 ± 5.67 | -0.75 ± 0.94 | <0.0001 | 0.0134 | -1.66, 4.20 |
|  | Day 84 | 78.78 ± 5.70 | -1.67 ± 0.96 | <0.0001 | <0.0001 | 2.54, 3.34 |
| **Hip circumference (cm)** | | | | | | |
| Placebo  (n= 30) | Baseline | 97.03 ± 5.67 | - | - | - | - |
|  | Day 42 | 96.92 ± 5.72 | -0.11 ± 1.07 | 0.5507 | - | - |
|  | Day 84 | 96.88 ± 5.54 | -0.15 ± 1.45 | 0.4932 | - | - |
| CL22209  100mg (n=30) | Baseline | 97.37 ± 5.85 | - | - | 0.8073 | -2.64, 3.32 |
|  | Day 42 | 96.58 ± 5.88 | -0.78 ± 0.95 | <0.0001 | 0.0148 | -2.66, 3.34 |
|  | Day 84 | 95.78 ± 5.88 | -1.59 ± 0.90 | <0.0001 | <0.0001 | -1.85, 4.05 |

A *p*-value < 0.05 indicates significance in intragroup (vs. baseline) or intergroup comparison (CL22209 vs. placebo) analyzed using ANCOVA followed by Bonferroni-Holm correction.

Supplementary Table S7: Serum endocrine factors and HOMA-IR (intention-to-treat analysis)

| Groups | Time of Evaluation | Mean ± SD | Change from baseline Mean ± SD | *p*‐value (vs. baseline) | *p*‐value (vs. placebo) | 95% CI (vs. placebo) |
| --- | --- | --- | --- | --- | --- | --- |
| **Luteinizing hormone (LH) (mIU/mL)** | | | | | | |
| Placebo  (n= 30) | Baseline | 12.13 ± 5.19 | - | - | - | - |
|  | Day 84 | 12.53 ± 5.95 | 0.40 ± 5.29 | 0.2775 | - | - |
| CL22209 100mg (n=30) | Baseline | 10.56 ± 3.52 | - | - | 0.1534 | -0.72, 3.86 |
|  | Day 84 | 11.29 ± 5.25 | 0.72 ± 5.63 | 0.4662 | 0.7925 | -1.66, 4.14 |
| **Follicle stimulating hormone (FSH) (mIU/mL)** | | | | | | |
| Placebo  (n= 30) | Baseline | 6.43 ± 1.83 | - | - | - | - |
|  | Day 84 | 6.82 ± 2.87 | 0.38 ± 2.45 | 0.5122 | - | - |
| CL22209 100mg (n=30) | Baseline | 6.24 ± 1.96 | - | - | 0.6889 | -0.79, 1.17 |
|  | Day 84 | 8.04 ± 4.84 | 1.80 ± 4.57 | 0.0042 | 0.1111 | -0.84, 3.28 |
| **LH to FSH ratio** | | | | | | |
| Placebo  (n= 30) | Baseline | 1.85 ± 0.42 | - | - | - | - |
|  | Day 84 | 1.83 ± 0.45 | -0.02 ± 0.54 | 0.6627 | - | - |
| CL22209 100mg (n=30) | Baseline | 1.74 ± 0.43 | - | - | 0.3462 | -0.11, 0.33 |
|  | Day 84 | 1.47 ± 0.44 | -0.27 ± 0.51 | 0.0002 | 0.0031 | 0.13, 0.59 |
| **Sex Hormone Binding Globulin (SHBG) (nmol/L)** | | | | | | |
| Placebo  (n= 30) | Baseline | 35.52 ± 13.12 | - | - | - | - |
|  | Day 84 | 31.69 ± 11.32 | -3.83 ± 14.68 | 0.0848 | - | - |
| CL22209 100mg (n=30) | Baseline | 34.99 ± 15.22 | - | - | 0.8833 | -6.81, 7.87 |
|  | Day 84 | 40.95 ± 14.18 | 5.97 ± 14.98 | 0.0036 | 0.0012 | 2.63, 15.89 |
| **Total testosterone (ng/mL)** | | | | | | |
| Placebo  (n= 30) | Baseline | 0.64 ± 0.46 | - | - | - | - |
|  | Day 84 | 0.65 ± 0.43 | 0.01 ± 0.22 | 0.8948 | - | - |
| CL22209 100mg (n=30) | Baseline | 0.63 ± 0.39 | - | - | 0.9329 | -0.21, 0.23 |
|  | Day 84 | 0.43 ± 0.28 | -0.20 ± 0.36 | <0.0001 | 0.0005 | 0.03, 0.41 |
| **Homeostatic Model Assessment for Insulin Resistance (HOMA-IR)** | | | | | | |
| Placebo  (n= 30) | Baseline | 3.19 ± 0.41 | - | - | - | - |
|  | Day 42 | 3.33 ± 0.52 | 0.15 ± 0.63 | 0.2529 | - | - |
|  | Day 84 | 3.28 ± 0.54 | 0.09 ± 0.65 | 0.6214 | - | - |
| CL22209 100mg (n=30) | Baseline | 3.25 ± 0.44 | - | - | 0.5644 | -0.16, 0.28 |
|  | Day 42 | 3.00 ± 0.49 | -0.26 ± 0.56 | 0.0032 | 0.0040 | 0.07, 0.59 |
|  | Day 84 | 2.80 ± 0.42 | -0.45 ± 0.57 | <0.0001 | <0.0001 | 0.23, 0.73 |

A *p*-value < 0.05 indicates significance in intragroup (vs. baseline) or intergroup comparison (CL22209 vs. placebo) analyzed using ANCOVA followed by Bonferroni-Holm correction.

**Supplementary Table S8:** Safety assessments- hematology and complete clinical biochemistry parameters

| Parameter | Evaluation Days | Group | | mean ± SD | *p*-value  (vs. baseline) | *p*-value  (vs. placebo) |
| --- | --- | --- | --- | --- | --- | --- |
| **Hematology** | | | | | | |
| Hemoglobin (g/dL) | Baseline | Placebo | | 11.83 ± 0.80 | - | - |
|  |  | CL22209 | | 11.90 ± 0.81 | - | NS |
|  | Day 84 | Placebo | | 11.92 ± 0.64 | 0.4308 | - |
|  |  | CL22209 | | 12.02 ± 0.62 | 0.0308 | NS |
| Platelet count  (10^9^/L) | Baseline | Placebo | | 289 ± 43 | - | - |
|  |  | CL22209 | | 288 ± 49 | - | NS |
|  | Day 84 | Placebo | | 286 ± 44 | 0.3410 | - |
|  |  | CL22209 | | 286 ± 47 | 0.7716 | NS |
| Erythrocyte sedimentation rate (ESR)  (mm/hr) | Baseline | Placebo | | 12.60 ± 1.81 | - | - |
|  |  | CL22209 | | 12.80 ± 1.77 | - | NS |
|  | Day 84 | Placebo | | 12.47 ± 1.41 | 0.4235 | - |
|  |  | CL22209 | | 12.62 ± 1.40 | 0.2268 | NS |
| Red blood cells  (million/cu.mm) | Baseline | Placebo | | 4.20 ± 0.48 | - | - |
|  |  | CL22209 | | 4.20 ± 0.35 | - | NS |
|  | Day 84 | Placebo | | 4.26 ± 0.43 | 0.2836 | - |
|  |  | CL22209 | | 4.19 ± 0.20 | 0.7631 | NS |
| White blood cells (cells/cu.mm) | Baseline | Placebo | | 7733 ± 791 | - | - |
|  |  | CL22209 | | 7780 ± 1078 | - | NS |
|  | Day 84 | Placebo | | 7777 ± 845 | 0.1868 | - |
|  |  | CL22209 | | 7934 ± 1060 | 0.1519 | NS |
| Neutrophil (%) | Baseline | Placebo | | 56.17 ± 7.36 | - | - |
|  |  | CL22209 | | 58.27 ± 6.31 | - | NS |
|  | Day 84 | Placebo | | 56.07 ± 5.99 | 0.8969 | - |
|  |  | CL22209 | | 57.90 ± 5.60 | 0.8817 | NS |
| Lymphocytes (%) | Baseline | Placebo | | 36.27 ± 7.61 | - | - |
|  |  | CL22209 | | 34.83 ± 6.34 | - | NS |
|  | Day 84 | Placebo | | 36.23 ± 6.42 | 0.9646 | - |
|  |  | CL22209 | | 34.97 ± 5.57 | 1.0000 | NS |
| Eosinophil (%) | Baseline | Placebo | | 6.17 ± 1.56 | - | - |
|  |  | CL22209 | | 5.50 ± 1.38 | - | NS |
|  | Day 84 | Placebo | | 6.13 ± 1.55 | 0.8230 | - |
|  |  | CL22209 | | 5.59 ± 1.32 | 1.0000 | NS |
| Monocytes (%) | Baseline | Placebo | | 1.40 ± 0.81 | - | - |
|  |  | CL22209 | | 1.40 ± 0.77 | - | NS |
|  | Day 84 | Placebo | | 1.57 ± 0.68 | 0.2018 | - |
|  |  | CL22209 | | 1.55 ± 0.74 | 0.2930 | NS |
| Basophils (%) | Baseline | Placebo | | 0.00 ± 0.00 | - | - |
|  |  | CL22209 | | 0.00 ± 0.00 | - | NS |
|  | Day 84 | Placebo | | 0.00 ± 0.00 | - | - |
|  |  | CL22209 | | 0.00 ± 0.00 | - | NS |
| **Blood biochemistry** | | | | | | |
| Fasting Blood Glucose (mg/dL) | Baseline | | Placebo | 90.57 ± 6.38 | - | - |
|  |  |  | CL22209 | 89.00 ± 7.91 | - | NS |
|  | Day 84 | | Placebo | 85.23 ± 5.78 | < 0.0001 | - |
|  |  |  | CL22209 | 83.69 ± 6.40 | < 0.0001 | NS |
| Creatinine  (mg/dL) | Baseline | | Placebo | 0.91 ± 0.13 | - | - |
|  |  |  | CL22209 | 0.91 ± 0.14 | - | NS |
|  | Day 84 | | Placebo | 0.91 ± 0.09 | 1.0000 | - |
|  |  |  | CL22209 | 0.92 ± 0.15 | 0.3259 | NS |
| Blood Urea Nitrogen (BUN)  (mg/dL) | Baseline | | Placebo | 11.57 ± 2.33 | - | - |
|  |  |  | CL22209 | 12.10 ± 2.28 | - | NS |
|  | Day 84 | | Placebo | 12.53 ± 3.37 | 0.0939 | - |
|  |  |  | CL22209 | 11.83 ± 1.47 | 0.7381 | NS |
| Blood Uric Acid (mg/dL) | Baseline | | Placebo | 4.38 ± 0.63 | - | - |
|  |  |  | CL22209 | 4.35 ± 0.71 | - | NS |
|  | Day 84 | | Placebo | 4.44 ± 0.60 | 0.2931 | - |
|  |  |  | CL22209 | 4.38 ± 0.72 | 0.4070 | NS |
| Sodium  (mmol/L) | Baseline | | Placebo | 140.43 ± 2.71 | - | - |
|  |  |  | CL22209 | 140.30 ± 3.65 | - | NS |
|  | Day 84 | | Placebo | 140.37 ± 2.37 | 0.8012 | - |
|  |  |  | CL22209 | 140.17 ± 3.44 | 0.6739 | NS |
| Potassium  (mmol/L) | Baseline | | Placebo | 4.11 ± 0.19 | - | - |
|  |  |  | CL22209 | 4.08 ± 0.18 | - | NS |
|  | Day 84 | | Placebo | 4.09 ± 0.14 | 0.2817 | - |
|  |  |  | CL22209 | 4.08 ± 0.14 | 0.8819 | NS |
| Alanine aminotransferase (ALT) (IU/L) | Baseline | | Placebo | 28.50 ± 5.71 | - | - |
|  |  |  | CL22209 | 26.57 ± 6.60 | - | NS |
|  | Day 84 | | Placebo | 29.43 ± 5.42 | 0.0576 | - |
|  |  |  | CL22209 | 27.66 ± 6.03 | 0.0591 | NS |
| Aspartate aminotransferase  (IU/L) | Baseline | | Placebo | 27.47 ± 4.27 | - | - |
|  |  |  | CL22209 | 27.07 ± 4.76 | - | NS |
|  | Day 84 | | Placebo | 27.53 ± 3.66 | 0.9286 | - |
|  |  |  | CL22209 | 26.24 ± 5.40 | 0.2638 | NS |
| Serum Alkaline Phosphatase (IU/L) | Baseline | | Placebo | 105.70 ± 13.74 | - | - |
|  |  |  | CL22209 | 112.93 ± 15.22 | - | NS |
|  | Day 84 | | Placebo | 106.60 ± 12.91 | 0.1503 | - |
|  |  |  | CL22209 | 112.83 ± 14.68 | 0.6102 | NS |
| Bilirubin (mg/dL) | Baseline | | Placebo | 0.70 ± 0.14 | - | - |
|  |  |  | CL22209 | 0.65 ± 0.14 | - | NS |
|  | Day 84 | | Placebo | 0.69 ± 0.13 | 0.3256 | - |
|  |  |  | CL22209 | 0.65 ± 0.11 | 1.0000 | NS |
| Albumin (g/dL) | Baseline | | Placebo | 4.02 ± 0.34 | - | - |
|  |  |  | CL22209 | 3.99 ± 0.32 | - | NS |
|  | Day 84 | | Placebo | 4.06 ± 0.32 | 0.0898 | - |
|  |  |  | CL22209 | 3.96 ± 0.28 | 0.5382 | NS |
| Creatinine kinase  (U/L) | Baseline | | Placebo | 72.23 ± 18.93 | - | - |
|  |  |  | CL22209 | 72.80 ± 22.74 | - | NS |
|  | Day 84 | | Placebo | 69.47 ± 15.35 | 0.1080 | - |
|  |  |  | CL22209 | 64.21 ± 15.90 | 0.0778 | NS |
| Lipid Profile | | | | | | |
| Low-density lipoprotein (LDL) (mg/dL) | Baseline | | Placebo | 114.47 ± 11.75 | - | - |
|  |  |  | CL22209 | 115.23 ± 8.44 | - | NS |
|  | Day 84 | | Placebo | 115.20 ± 10.51 | 0.3336 | - |
|  |  |  | CL22209 | 117.28 ± 8.30 | 0.1405 | NS |
| High-density lipoprotein (HDL) (mg/dL) | Baseline | | Placebo | 43.63 ± 2.19 | - | - |
|  |  |  | CL22209 | 44.50 ± 1.98 | - | NS |
|  | Day 84 | | Placebo | 44.07 ± 2.50 | 0.1868 | - |
|  |  |  | CL22209 | 44.86 ± 1.85 | 0.2157 | NS |
| Very low-density lipoprotein (VLDL) (mg/dL) | Baseline | | Placebo | 27.60 ± 2.75 | - | - |
|  |  |  | CL22209 | 28.13 ± 3.03 | - | NS |
|  | Day 84 | | Placebo | 27.90 ± 2.62 | 0.1303 | - |
|  |  |  | CL22209 | 29.00 ± 2.71 | 0.1110 | NS |
| Triglycerides (mg/dL) | Baseline | | Placebo | 139.20 ± 13.35 | - | - |
|  |  |  | CL22209 | 140.30 ± 15.55 | - | NS |
|  | Day 84 | | Placebo | 140.73 ± 10.32 | 0.1900 | - |
|  |  |  | CL22209 | 144.48 ± 10.24 | 0.1417 | NS |
| Total cholesterol (mg/dL) | Baseline | | Placebo | 186.23 ± 13.88 | - | - |
|  |  |  | CL22209 | 192.10 ± 10.76 | - | NS |
|  | Day 84 | | Placebo | 187.33 ± 13.32 | 0.3472 | - |
|  |  |  | CL22209 | 190.66 ± 9.53 | 0.5455 | NS |

The data at the screening visit are considered as the baseline data. NS, not significant. A *p*-value < 0.05 is considered statistically significant. Intragroup comparison (vs. baseline) was analyzed using a paired t-test, and difference between the groups (placebo vs. CL22209) at screening and the end of the study (day 84), analyzed using one-way ANOVA. At baseline: Placebo (n = 30), CL22209 (n = 30), day 84: Placebo (n = 30), CL22209 (n = 29).
